# SCODA: A Low-Cost Prehabilitation Strategy to Improve Outcomes After Cytoreductive Surgery in a Low-Resource Setting

**DOI:** 10.3390/cancers17223687

**Published:** 2025-11-18

**Authors:** Amine Souadka, Lina Alami, Zakaria Elmouatassim, Oumayma Lahnaoui, Yassine El Bouazizi, Sabrillah Echiguer, Oussama Ssouni, Ayman El Fassi, Abdelilah Ghannam, Zakaria Houssain Belkhadir, Brahim El Ahmadi

**Affiliations:** 1 Surgical Oncology Department, National Institute of Oncology, University Mohammed V in Rabat, Rabat 10100, Morocco; lina_alami@um5.ac.ma (L.A.); zakaria.elmouatassim@um5s.net.ma (Z.E.); oumayma.lah@gmail.com (O.L.); elbouazizi.yassine@gmail.com (Y.E.B.); sabrillah.echiguer@um5r.ac.ma (S.E.); 2 Equipe de Recherche en Oncologie Translationnelle (EROT), Faculty of Medicine and Pharmacy, Mohammed V University in Rabat, Rabat 10100, Morocco; 3 Intensive Care Department, National Institute of Oncology, University Mohammed V in Rabat, Rabat 10100, Morocco; o.ssouni@um5r.ac.ma (O.S.); ayman.elfassi@gmail.com (A.E.F.); abdelilah.ghannam@gmail.com (A.G.); z.belkhadir@um5r.ac.ma (Z.H.B.); b.elahmadi@um5r.ac.ma (B.E.A.)

**Keywords:** prehabilitation, cytoreductive surgery, HIPEC, low- and middle-income countries (LMICs), postoperative complications

## Abstract

**Background**: Cytoreductive surgery (CRS) with or without hyperthermic intraperitoneal chemotherapy (HIPEC) remains the standard of care for peritoneal surface malignancies but carries significant postoperative risks. In low- and middle-income countries (LMICs), the challenge is magnified by limited access to high-cost enhanced recovery programs. The SCODA (Surgical Complication Optimization through Diet and Activity) program was developed as a low-cost prehabilitation intervention to improve outcomes in resource-constrained settings. **Methods**: This retrospective cohort study included 169 patients undergoing CRS ± HIPEC at a single academic center in Morocco between 2015 and 2023. Patients treated before SCODA implementation (pre-SCODA group, *n* = 83) were compared to those enrolled in the SCODA program (SCODA group, *n* = 86). SCODA included oral iron supplementation, a protein-rich food-based diet, and progressive walking sessions over 90 days. Perioperative outcomes, including pulmonary complications, major morbidity (Clavien–Dindo ≥ 3b), transfusions, ICU stay >3 days, and 90-day mortality, were analyzed using univariate and multivariate logistic regression models. **Results**: The SCODA group had significantly fewer pulmonary complications (2% vs. 13%, *p* = 0.008), reduced major morbidity (9% vs. 21%, *p* = 0.031), fewer transfusions (8% vs. 20%, *p* = 0.024), and shorter ICU stays (median 1.5 vs. 5 days, *p* < 0.001). Ninety-day mortality was also lower in the SCODA group (5.8% vs. 12.4%, *p* = 0.046). SCODA participation remained an independent protective factor in multivariate analyses for all endpoints except major complications. **Conclusions**: The SCODA program is a feasible and effective prehabilitation strategy for improving surgical outcomes after CRS/HIPEC in LMICs. Its low-cost, food-based, and activity-centered design may support broader implementation in resource-limited environments and inform future perioperative care policies in oncology.

## Simple Summary

Patients undergoing cytoreductive surgery with or without HIPEC face high rates of postoperative complications, especially in low- and middle-income countries where access to costly enhanced recovery programs is limited. To address this challenge, we developed SCODA, a simple and low-cost prehabilitation program based on daily walking, high-protein nutrition using affordable local foods, and routine oral iron supplementation over 90 days before surgery. In this study including 169 patients, SCODA significantly reduced pulmonary complications, major morbidity, transfusion needs, ICU stay, and 90-day mortality compared with standard care. These findings show that an accessible, food-based and activity-centered approach can strengthen perioperative care in resource-constrained settings and may serve as a scalable model for improving outcomes in complex oncologic surgery.

## 1. Introduction

Cytoreductive surgery (CRS), with or without hyperthermic intraperitoneal chemotherapy (HIPEC), has become the standard of care for selected patients with peritoneal surface malignancies, including colorectal, gastric, and ovarian cancers, as well as pseudomyxoma peritonei and peritoneal mesothelioma [1]. Despite offering curative potential, CRS/HIPEC remains a complex procedure associated with high postoperative morbidity and prolonged recovery, largely influenced by the patient’s baseline physiological status and the extent of surgical resection required [2,3].

Prehabilitation has emerged as a strategy to enhance surgical resilience by optimizing patients’ physical, nutritional, and psychological status prior to major oncologic surgery [4]. Evidence supports its benefits in reducing complications, improving quality of life, and shortening recovery time [5,6,7,8]. However, most of these programs were developed in high-resource settings and depend on specialized care structures that are often unavailable in low- and middle-income countries (LMICs).

Enhanced Recovery After Surgery (ERAS) programs have demonstrated efficacy in improving outcomes in CRS/HIPEC [9,10,11]. Yet, their reliance on costly interventions and advanced perioperative infrastructure significantly limits their implementation in LMICs, where access to such resources remains a challenge.

To address these constraints, the National Institute of Oncology in Rabat, Morocco, implemented the SCODA program (Surgical Complication Optimization through Diet and Activity) in 2020. This low-cost initiative comprises structured physical activity, protein-rich dietary modifications, and oral iron supplementation. This study evaluates the SCODA program’s impact on perioperative outcomes—including complications, transfusions, and ICU utilization—highlighting its potential as a pragmatic model for surgical optimization in resource-limited settings.

## 2. Methods

### 2.1. Study Design

This retrospective cohort study was conducted at the National Institute of Oncology in Rabat, a tertiary referral center and academic teaching hospital. The SCODA prehabilitation program was launched in January 2020. To evaluate its impact, we compared postoperative outcomes between two groups: patients who underwent CRS with or without HIPEC from 2015 to 2019 without prehabilitation (pre-SCODA group) and those treated from 2020 onward after completing the SCODA program (SCODA group). The study was conducted in accordance with the STROBE statement for observational studies. Ethical approval was not required for this study, in line with Moroccan Law 28/13 (article 2), which exempts retrospective analyses from review under specific conditions. All procedures followed the ethical standards of the Declaration of Helsinki (1964) and its later amendments. Written informed consent was obtained from all participants.

### 2.2. Patient Eligibility and Selection

All consecutive patients diagnosed with peritoneal carcinomatosis from colorectal cancer (CRC), gastric cancer (GC), pseudomyxoma peritonei (PMP), or advanced-stage ovarian cancer (OvC stage IIIB–IV) who underwent cytoreductive surgery, with or without HIPEC, were eligible for inclusion. In our setting, HIPEC is considered mainly for PMP and colorectal carcinomatosis with limited disease burden (PCI < 20) and good performance status. Gastric carcinomatosis and ovarian cancer and extensive disease were generally managed without HIPEC due to uncertain benefit and resource limitations. Patients in the SCODA group were scheduled for surgery at least three months in advance to allow completion of the prehabilitation program. A multidisciplinary team, including surgical oncologists, nutritionists, physiotherapists, and anesthesiologists, ensured consistent program delivery, optimization of comorbidities (e.g., antihypertensives, glycemic control, and monitored adherence. Patients unable to perform moderate exertion, such as climbing two to three flights of stairs without undue fatigue [12], were deemed unfit for CRS/HIPEC due to concerns about their physiological reserve. Those who failed to comply with core prehabilitation components were excluded from the surgical pathway and offered alternative oncologic management.

### 2.3. Intervention: The SCODA Program

**Figure 1:**
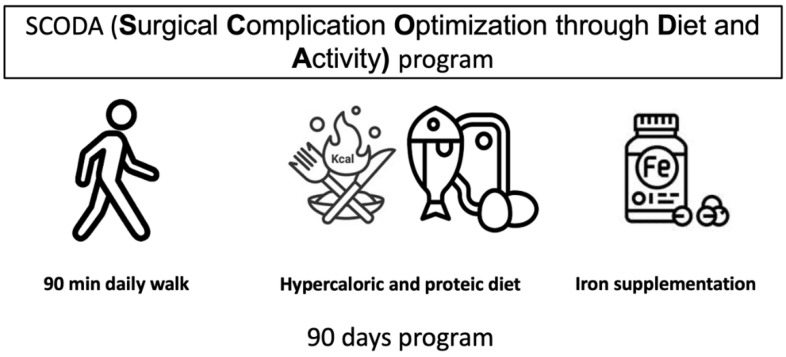
Overview of the SCODA (Surgical Complication Optimization through Diet and Activity) Program. Caption: The SCODA program is a 90-day, low-cost prehabilitation intervention designed for patients undergoing cytoreductive surgery with or without HIPEC. It includes three core components: (1) daily 90 min walking sessions to improve cardiopulmonary fitness, (2) a hypercaloric and protein-rich diet to address preoperative malnutrition and sarcopenia, and (3) oral iron supplementation (ferrous sulfate 325 mg/day) to optimize hemoglobin levels and reduce transfusion requirements. The program is adapted for feasibility in low- and middle-income countries.

The SCODA (Surgical Complication Optimization through Diet and Activity) program consisted of a structured, 90-day multimodal prehabilitation regimen tailored to resource-limited settings. It included three core interventions (Figure 1):

1. **Iron supplementation** (ferrous sulfate 325 mg/day) aimed to correct preoperative anemia, a prevalent and modifiable risk factor for transfusions, particularly in LMIC contexts. The 90-day duration corresponded to the erythropoiesis cycle, allowing time for hematologic response.
2. **Food-based nutritional prehabilitation**: (1.5–2 g of protein/kg/day) Nutritional counseling prioritized low-cost, locally available protein sources, including legumes (lentils, chickpeas), eggs, and sardines combined with caloric enhancers such as olive oil and nuts. While patients were encouraged to incorporate additional protein sources when feasible, the core dietary recommendations were designed to remain accessible in low-resource settings.
3. **Graded walking regimen**, progressively increasing from 30 to 90 min daily over three weeks, was prescribed to improve cardiopulmonary fitness and reduce the risk of pulmonary complications.

Patients were supervised through weekly counseling sessions and safety assessments. Adherence was reinforced with clear guidance and written instructions. No adverse events related to iron supplementation or physical activity were reported during the study period.

### 2.4. ERAS Protocol and Pre-Anesthesia Optimization

All patients, irrespective of group allocation, received perioperative care based on an Enhanced Recovery After Surgery (ERAS) protocol, adapted to the realities of low-resource environments. The protocol was streamlined to emphasize essential interventions with high impact and minimal cost, ensuring feasibility within LMICs.

#### 2.4.1. Cardiopulmonary Optimization

Patients with high anesthetic risk (ASA ≥ 3) underwent systematic preoperative evaluation, including echocardiography and spirometry. For those with limited functional reserve, targeted physiotherapy and respiratory training were implemented. Previous evidence suggests that such interventions reduce ICU length of stay by approximately 30% in the CRS/HIPEC population [13].

#### 2.4.2. Nutritional and Hematologic Preparation

Iron supplementation followed the SCODA protocol. Preoperative hemoglobin thresholds for transfusion were standardized (Hb < 8 g/dL). Patients also received oral carbohydrate loading (glucose-rich solution) six hours before surgery to reduce insulin resistance and improve metabolic response.

#### 2.4.3. Psychological Counseling

All patients underwent at least one structured preoperative session led primarily by the operating surgeon to explain the goals and sequence of ERAS care and support by the trained healthcare team. Psychological counseling was provided as part of preoperative care, emphasizing key points on stress management and setting realistic surgical expectations. Due to resource constraints, formal outcome scales were not used. Particular attention was paid to the concept of RIOT (Return to Intended Oncologic Therapy), emphasizing the relationship between perioperative recovery and timely initiation of adjuvant treatments. This dialogue was designed to enhance compliance and reduce perioperative anxiety.

### 2.5. Intraoperative Management and Postoperative Phase

#### 2.5.1. Hemodynamic Monitoring

Goal-Directed Fluid Therapy (GDFT) was implemented for all patients using non-invasive cardiac output monitoring. This approach aimed to optimize fluid balance, maintain tissue perfusion, and minimize the risk of fluid overload—an important concern in CRS/HIPEC, where fluid shifts are pronounced.

#### 2.5.2. Anesthetic Protocol

A standardized anesthetic regimen combining epidural and general anesthesia was used to enhance intraoperative pain control and reduce postoperative opioid consumption. This strategy has been associated with improved gut motility and shorter time to ambulation, both key targets of enhanced recovery.

#### 2.5.3. Surgical Safety Measures

A mandatory intraoperative “Safe Anastomosis Checklist” was employed before completion of intestinal anastomoses to ensure optimal perfusion, tension-free anastomosis, and patient hemodynamic stability. This checklist, previously validated in our institution [13], was linked to a lower incidence of anastomotic leaks and urgent reinterventions.

#### 2.5.4. Early Mobilization and Vigilance

Patients were encouraged to sit on the bed on POD 0 and ambulate within 24 h of surgery (POD 1), facilitated by the prehabilitation program’s prior conditioning. Postoperatively, daily multidisciplinary rounds involving both surgeons and anesthesiologists ensured close monitoring for complications, enabling early detection and intervention. For instance, re-laparotomy was promptly undertaken for any complication meeting or exceeding Clavien–Dindo grade 3b criteria.

### 2.6. Outcome Measures

The primary outcomes of this study were postoperative complications, stratified according to the Clavien–Dindo classification system (grade I–V) [14], with a particular focus on grade ≥ 3b complications requiring surgical, endoscopic, or radiological intervention under general anesthesia. The ≥3b threshold effectively captures major complications requiring surgical or other significant interventions under general anesthesia [15]. Additional primary endpoints included the incidence of pulmonary complications (e.g., pneumonia, respiratory failure), transfusion requirements (≥1 unit of red blood cells during the perioperative period), ICU stay longer than 3 days, and 90-day postoperative mortality.

Secondary outcomes included adherence to the SCODA program, evaluated through weekly checklists and review of preoperative follow-up notes, with physical activity self-reported and cross-checked during visits, iron administration confirmed via prescription records, and dietary counseling reinforced and documented in patient logs; patient-reported satisfaction with the intervention; and pre- to postoperative changes in hemoglobin and albumin levels. Functional status evolution (based on ECOG score) was also assessed in the SCODA group by our multidisciplinary team, reflecting the impact of physical activity and nutritional support on postoperative recovery.

These outcomes were selected to reflect both clinical efficacy and feasibility of the SCODA program in a real-world, resource-constrained setting.

### 2.7. Data Collection and Analysis

Data were extracted from prospectively maintained institutional databases and verified against individual medical records. Demographic variables included age, sex, BMI, cancer origin, ECOG performance status, ASA score, and receipt of neoadjuvant therapy. Surgical details captured included the number of anastomoses, presence of HIPEC, and Peritoneal Cancer Index (PCI) score. Laboratory values such as pre- and postoperative hemoglobin and albumin levels were collected to assess the nutritional and hematologic impact of the SCODA intervention.

Continuous variables were reported as means with standard deviations or medians with interquartile ranges, depending on normality assessed via the Shapiro–Wilk test. Categorical variables were presented as frequencies and percentages. Between-group comparisons (SCODA vs. pre-SCODA) were performed using the chi-square or Fisher’s exact test for categorical variables and the independent samples *t*-test or Mann–Whitney U test for continuous variables, as appropriate. To ensure clinical relevance and statistical robustness, our multivariate model was carefully constructed by selecting covariates with strong clinical significance and univariate *p*-values less than 0.10, utilizing transparent and interpretable logistic regression.

Univariate logistic regression analyses were conducted to identify associations between covariates (e.g., age, sex, PCI, ECOG, ASA, SCODA participation) and binary outcomes (e.g., transfusions, major complications, ICU > 3 days, mortality). Multivariate logistic regression models were then constructed for each outcome with adequate event counts (typically ≥ 10 events per variable), adjusting for clinically relevant confounders and variables with *p* < 0.10 in univariate analysis. Although HIPEC and operative duration were clinically meaningful, they were more frequent in the SCODA group and independently associated with higher surgical risk. Including them could have artificially amplified the protective effect of SCODA. To avoid overadjustment, these variables were excluded, and surgical complexity was instead represented by the Peritoneal Cancer Index (PCI), a validated measure of intra-abdominal tumor burden and operative extent. For outcomes with low event counts, parsimonious models (max 2–3 covariates) were used to reduce the risk of overfitting. Model performance was assessed via Hosmer–Lemeshow goodness-of-fit testing. Statistical significance was set at a two-tailed *p*-value < 0.05. Analyses were performed using IBM SPSS Statistics (version 27.0; IBM Corp., Armonk, NY, USA).

## 3. Results

### 3.1. Patient Demographics and Baseline Characteristics

A total of 169 patients were included, with 83 in the pre-SCODA group and 86 in the SCODA group. The mean age was 54.4 years (SD 10.5) in the pre-SCODA group and 56.3 years (SD 9.1) in the SCODA group. BMI was similar between groups (23.4 vs. 22.0 kg/m^2^). Median PCI was 10 in both groups, with a slightly higher mean PCI in the SCODA group (11 vs. 10). ECOG scores of 2–3 were reported in 34% of the pre-SCODA group and 40% of the SCODA group. ASA scores of 2–3 were more frequent in the SCODA group (84% vs. 67%). Neoadjuvant therapy was administered in 29 patients (33%) in the SCODA group compared to 22 (23%) in the pre-SCODA group. Primary tumor types and other baseline variables are summarized in Table 1 and Table S1.

### 3.2. Perioperative Outcomes

**Figure 2:**
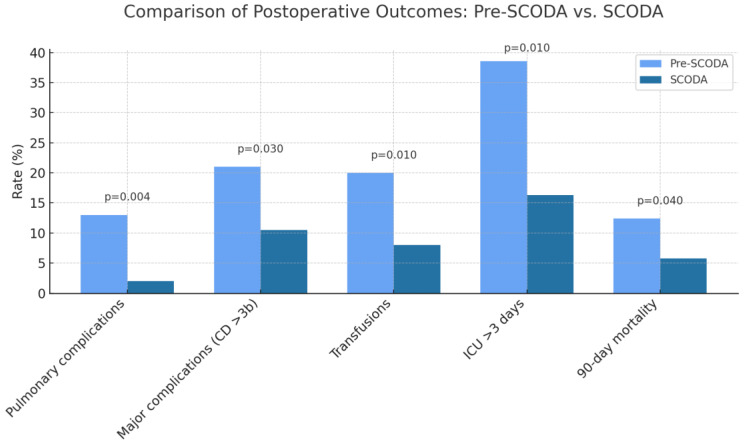
Comparison of postoperative complication rates, transfusion requirements, prolonged ICU stays, and 90-day mortality between patients managed before and after SCODA program implementation. Significant reductions in all outcomes are observed in the SCODA group.

The SCODA group showed significant improvements across several perioperative indicators. Pulmonary complications occurred in only 2 patients (2%) in the SCODA group versus 11 (13%) in the pre-SCODA group. (Table 2) Major complications (Clavien–Dindo ≥ 3b) were less frequent in the SCODA group (9% vs. 21%). Median ICU stay was reduced from 5 days (pre-SCODA) to 1.5 days (SCODA). Perioperative transfusions decreased from 20% in the pre-SCODA group to 8% in the SCODA group. No adverse events related to iron supplementation or physical activity were reported during the study period. The 90-day mortality rate was lower in the SCODA group (5.8%) compared to the pre-SCODA group (12.4%). These outcomes are illustrated in Figure 2.

### 3.3. Multivariate Analysis

Multivariate regression confirmed that SCODA participation was independently associated with a significantly reduced risk of pulmonary complications (adjusted OR 0.26; 95% CI, 0.10–0.64; *p* = 0.004). Increasing PCI was also associated with elevated pulmonary risk (adjusted OR 1.21 per point; 95% CI, 1.08–1.36; *p* = 0.001) (Table 3).

SCODA participation showed a non-significant trend toward lower rates of major complications (adjusted OR 0.56; 95% CI, 0.28–1.13; *p* = 0.106). However, both PCI (adjusted OR 1.15; 95% CI, 1.05–1.26; *p* = 0.002) and age (adjusted OR 1.04 per year; 95% CI, 1.00–1.08; *p* = 0.043) remained significant predictors of severe postoperative morbidity.

Regarding transfusions, SCODA participation was independently associated with a markedly lower likelihood of requiring blood products (adjusted OR 0.16; 95% CI, 0.07–0.38; *p* = 0.005). PCI again correlated with increased transfusion need (adjusted OR 1.13; 95% CI, 1.03–1.25; *p* = 0.014), while preoperative hemoglobin did not (adjusted OR 1.10; 95% CI, 0.86–1.43; *p* = 0.415).

SCODA participation significantly reduced the odds of prolonged ICU stay (>3 days) (adjusted OR 0.36; 95% CI, 0.16–0.81; *p* = 0.014). Both higher PCI (adjusted OR 1.20; 95% CI, 1.08–1.33; *p* = 0.001) and ECOG 2–3 (adjusted OR 1.95; 95% CI, 1.31–2.90; *p* = 0.001) were associated with longer ICU stays.

Finally, SCODA participants had a significantly lower risk of 90-day mortality (adjusted OR 0.41; 95% CI, 0.20–0.84; *p* = 0.014), with PCI again emerging as an independent predictor (adjusted OR 1.19; 95% CI, 1.09–1.31; *p* = 0.005).

## 4. Discussion

### 4.1. A Pragmatic Prehabilitation Model for LMIC Settings

In this pilot study, the SCODA program comprising a 90 min daily walk, a high-protein diet, and iron supplementation over 90 days prior to CRS/HIPEC, proved feasible, with high adherence and favorable postoperative outcomes. These findings not only support existing global evidence on the benefits of prehabilitation but also reinforce the feasibility of implementing such programs in LMICs.

SCODA represents an adaptable version of enhanced recovery pathways such as ERAS, specifically tailored to the constraints of LMIC settings. While ERAS incorporates multimodal interventions aimed at optimizing recovery and shortening hospital stays [16], its dependence on high-resource infrastructure often limits its uptake in under-resourced environments. In contrast, SCODA focuses on accessible interventions, such as daily walking and nutritional support [17], that achieve comparable improvements in outcomes without the need for advanced technology or costly logistics.

### 4.2. Physical Activity and Duration of Prehabilitation

Physical activity has emerged as a cornerstone of effective prehabilitation. Most programs reported in the literature span fewer than six weeks, aiming to optimize functional capacity in a limited timeframe [18]. While these short-term interventions have demonstrated benefits, longer programs may offer sustained physiological improvements, though their impact on long-term outcomes such as survival or recurrence remains insufficiently studied [19].

High-intensity interval training (HIIT), commonly used to enhance aerobic capacity, alternates short bursts of effort with rest. However, systematic reviews suggest that HIIT does not significantly outperform moderate-intensity activity in improving peak oxygen uptake (VO_2_peak) or in reducing postoperative complications [20]. Furthermore, many existing trials were underpowered to assess clinical outcomes, despite high adherence rates [20].

The SCODA program prioritized low- to moderate-intensity daily walking over a prolonged period of 90 days. This longer duration aligns better with the realities of LMICs, where patients may present earlier and have more flexibility to engage in preoperative conditioning. Our findings indicate a strong adherence rate of 80% for physical activity, supporting the feasibility of sustained, low-resource exercise interventions in surgical oncology pathways.

### 4.3. Nutritional Support and Protein Intake

Adequate nutritional support is a critical determinant of postoperative recovery and has been consistently associated with improved surgical outcomes. Malnutrition, present in more than one-third of patients undergoing cytoreductive surgery, has been linked to increased risks of infectious complications, impaired wound healing, and prolonged recovery [21]. Early nutritional interventions, even as short as one week before surgery, have been shown to reduce major complications—including respiratory, renal, and cardiac events—by up to 50% [22].

Prehabilitation strategies often incorporate carbohydrate loading; however, protein intake remains the cornerstone of metabolic support, particularly in oncologic lipolysis [23,24]. Studies suggest that an intake of 1.2 to 1.5 g of protein per kilogram of body weight per day is necessary to counteract catabolism and promote tissue repair [25,26]. Patients who meet these protein targets have been observed to regain functionality more rapidly and experience fewer postoperative complications [27].

In the Moroccan context, our program addressed a unique behavioral observation: following a cancer diagnosis, patients tend to reduce their protein intake by nearly 50% [28]. This finding underscored the importance of culturally adapted dietary counseling focused on high-protein nutrition. In our cohort, the SCODA intervention led to preoperative prealbumin levels comparable to those reported in international studies. Notably, the work by Díaz-Feijoo et al. showed significantly improved prealbumin levels in the prehabilitation group compared to controls, validating the link between protein supplementation and nutritional status prior to surgery [27]. The high adherence rate observed in our cohort further supports the feasibility of such an intervention in low-resource settings.

### 4.4. Iron Supplementation and Transfusion Avoidance

Anemia is a frequent preoperative concern in patients undergoing cytoreductive surgery, with transfusion rates reaching up to 77% and an average of three red blood cell (RBC) units administered per patient [29]. This poses a major challenge in low- and middle-income countries, where blood supply is often scarce and unpredictable. Optimizing hemoglobin levels prior to surgery through iron supplementation is therefore a strategic necessity in such settings.

Several studies have demonstrated the efficacy of intravenous iron therapy in correcting iron deficiency anemia and reducing transfusion requirements, particularly when administered more than seven days before surgery [30]. Beyond improving hematologic parameters, this intervention has also been associated with shorter hospital stays and lower rates of postoperative complications.

Although episodic blood shortages exist in our national context, the reduction in transfusions observed in the SCODA group was not related to supply constraints. All transfusions were administered according to predefined clinical thresholds, indicating a clear effect of prehabilitation on transfusion requirements. In our cohort, the SCODA program incorporated routine preoperative iron supplementation as a core component. This led to a marked reduction in the proportion of patients requiring perioperative transfusions, from 17 cases to 7, corresponding to a 58.8% decrease (*p* = 0.047). This improvement is not only clinically significant but also highly relevant in the context of constrained blood bank resources. By proactively addressing anemia, the SCODA protocol enhanced patient safety and contributed to more efficient perioperative care without overburdening the healthcare system.

### 4.5. Psychological Support and Program Adherence

Psychological preparation is often overlooked in surgical pathways, yet it plays a pivotal role in patient engagement and adherence to prehabilitation protocols. In our experience, counseling sessions were essential to helping patients understand the rationale and expected benefits of the SCODA program. This support appeared to enhance motivation and consistency, particularly for lifestyle components such as walking and dietary adjustments.

While robust evidence linking psychological support directly to improved postoperative outcomes remains limited, its indirect influence through better adherence is increasingly recognized [27]. In our setting, where health literacy and access to information can be variable, the integration of simple, in-person educational interactions proved both feasible and impactful.

By fostering a sense of agency and reducing anxiety, psychological support likely contributed to the high adherence rates observed across the three SCODA components. Although further research is needed to quantify this effect, it remains a key enabler of prehabilitation success in real-world LMIC contexts.

### 4.6. Combined Impact on Postoperative Outcomes

When delivered as a package, the components of the SCODA program appear to exert a synergistic effect on surgical recovery. While each intervention, physical activity, nutritional support, iron supplementation, has individually demonstrated benefits, their integration likely amplifies the physiological resilience of patients undergoing CRS/HIPEC.

In our analysis, the SCODA program was associated with a 44% reduction in the odds of major complications (Clavien–Dindo ≥ 3b), although this finding did not reach statistical significance. Despite this, the trend suggests that prehabilitation may help mitigate the systemic stress of extensive surgery, potentially smoothing the postoperative course.

Additionally, established risk factors such as high PCI were reaffirmed in our dataset as strong predictors of adverse outcomes, including complications, prolonged ICU stay, and postoperative mortality [30]. Age also emerged as a modest but significant predictor of severe complications (adjusted OR: 1.04, *p* = 0.043), echoing findings from prior studies that associate aging with diminished physiological reserves [31,32,33].

Functional status, assessed via the ECOG performance score, was a particularly strong determinant of postoperative resilience. Patients with ECOG 2–3 had nearly double the risk of requiring extended ICU care (OR: 1.95, *p* = 0.001), aligning with literature linking reduced baseline function to higher perioperative morbidity and mortality [34]. To address patients whose physiological status do not fit the criteria to undergo CRS (ECOG ≥ 3, ASA IV, Hb < 8 g/dL), they are not scheduled for CRS immediately. Instead, they are offered the SCODA program for 4 to 6 weeks, and reassessed before surgery. This individualized approach aligns with safe patient selection in resource-limited contexts.

Taken together, these findings suggest that the SCODA program offers meaningful benefit, particularly when implemented in patients with modifiable risk profiles. Its role in buffering surgical stress highlights its relevance not only for outcome improvement, but also for resource planning in constrained environments.

### 4.7. Reducing Pulmonary Complications and Mortality

Pulmonary complications remain a significant cause of postoperative morbidity following CRS/HIPEC. While evidence from multimodal prehabilitation programs has shown variable impact on respiratory outcomes [35], our findings suggest a substantial protective effect associated with SCODA. The observed 81.8% reduction in pulmonary events is noteworthy and likely reflects improved preoperative conditioning, even though the precise mechanism remains multifactorial and difficult to isolate.

The extended duration of the SCODA intervention, spanning 90 days, may have contributed to this benefit. Unlike conventional programs, which often last 2 to 4 weeks [36], SCODA provided patients with a longer adaptation period, potentially enhancing cardiorespiratory function and physical resilience. This extended timeline, combined with structured walking routines and simplified respiratory training, may explain the lower complication rates.

In addition to respiratory benefits, the program also demonstrated a 59% reduction in 90-day mortality [13], a result that reinforces the overall impact of prehabilitation in high-risk surgical oncology. Although causality cannot be definitively established, this decline likely stems from the cumulative effect of fewer transfusions, shorter ICU stays, and improved nutritional and functional status.

To our knowledge, SCODA is the first comprehensive, adaptable prehabilitation program designed specifically for CRS/HIPEC in LMICs. By addressing multiple modifiable risk factors preoperatively, it offers a pathway toward not only better clinical outcomes, but also more sustainable surgical care. In particular, the reductions in mortality, ICU burden, and transfusion requirements speak to the broader value of prehabilitation as a public health strategy in settings where healthcare resources are limited and postoperative complications can overwhelm capacity

### 4.8. Implementation and Sustainability

The SCODA program was designed for easy integration into routine care using existing hospital staff, with no need for additional infrastructure. Educational components, focused on walking and nutrition, were delivered through simple in-person sessions and printed materials, ensuring feasibility even in non-academic centers.

To enhance scalability, low-tech tools such as SMS reminders or mobile apps could support patient adherence without increasing costs. Broader implementation would benefit from early engagement of stakeholders, including hospital leadership and perioperative teams, as well as cultural adaptation of dietary advice.

SCODA is a flexible model rather than a rigid protocol. Frameworks like RE-AIM could support national dissemination while preserving fidelity. Its low-cost design makes it particularly suited for integration into standard perioperative care in LMICs.

### 4.9. Policy Implication

Given its feasibility, affordability, and demonstrated benefits, the SCODA program may serve as a model for national surgical guidelines on perioperative care in LMICs. Its adoption could bridge the gap in surgical outcomes between resource-limited and high-income settings.

### 4.10. Challenges and Lessons Learned

Several operational lessons emerged during SCODA’s implementation. Maintaining adherence to walking and dietary routines required continuous patient education and structured follow-up. The program’s success was closely tied to the commitment of the care team in reinforcing behavioral change.

Scaling beyond a single center will require coordinated efforts across institutions and regions. Cultural adaptation proved essential, particularly in aligning dietary recommendations with local habits and patient beliefs. These insights emphasize the importance of flexibility and contextualization when deploying prehabilitation programs in LMIC settings.

### 4.11. Limitations

This study has several limitations. The retrospective and single-center design may limit the generalizability of the findings to other settings. Many ERAS studies originate from high-income countries where resources differ from our setting. In our LMIC context, constraints such as staffing, equipment, patient socioeconomic barriers, and blood shortage, may limit implementation fidelity. Therefore, while our findings support the feasibility of maladapted ERAS elements locally, caution is needed for future implementations when extrapolating these results to other settings with different resource profiles. In terms of data collection methods, differences between before and after the implementation of the SCODA program could introduce information bias. While cause-specific mortality data were available, the limited number of events precluded a meaningful stratified analysis. Mortality was considered a global indicator of perioperative safety, consistent with prior studies. Future research should explore detailed cause-of-death pathways in larger prospective cohorts. In addition, the relatively small number of patients and outcome events limited the power of regression analyses and the number of variables that could be reliably assessed. Another limitation is the heterogeneity of tumor origins in our cohort (colorectal, gastric, ovarian, and pseudomyxoma peritonei). Although we ensured a balanced distribution between groups (Table 1), and the sample size was limited, no further stratified or adjusted analysis by tumor type was performed.

Assessment of nutritional improvement was also challenging. The presence of ascites in many patients with peritoneal carcinomatosis may have biased BMI measurements, making them an unreliable marker of nutritional gain. More accurate measures, such as muscle mass measurement, handgrip strength, or skeletal muscle index, could have provided better insight into sarcopenia [37], but were not routinely available due to resource limitations, including restricted access to functional testing and CT-based body composition analysis, which could be explored in future research.

Although HIPEC and operative duration are important factors influencing postoperative outcomes, we intentionally excluded them from our multivariate model. These variables were more frequent in the SCODA group and are inherently associated with higher surgical complexity and complication rates. Including them could have paradoxically strengthened the observed protective effect of SCODA, introducing bias in the interpretation. Instead, we relied on the PCI score, a validated proxy for surgical complexity, to account for intraoperative risk. This approach, though conservative, was aimed at isolating the true contribution of the prehabilitation program without confounding from procedure-related factors.

Finally, the absence of long-term follow-up data limits our ability to assess outcomes such as sustained functional recovery, quality of life, and the economic impact of the intervention. Prospective, multicenter studies using standardized, evidence-based prehabilitation protocols and tailored digital health tools are needed to validate these findings. Future research should also explore cost-effectiveness and implementation strategies across diverse healthcare systems, particularly in low- and middle-income countries.

## 5. Conclusions

The SCODA program demonstrates that a pragmatic, low-cost prehabilitation approach, centered on daily walking, protein-rich nutrition, and iron supplementation, can meaningfully improve perioperative outcomes in patients undergoing CRS with or without HIPEC. Despite being implemented in a resource-limited setting, SCODA significantly reduced pulmonary complications, ICU stays, transfusion needs, and 90-day mortality.

Its simplicity, high adherence, and adaptability make it a scalable model for surgical oncology in low- and middle-income countries. Beyond clinical efficacy, SCODA addresses systemic constraints, offering a cost-effective pathway to improve surgical safety and optimize resource utilization.

Future multicenter trials are warranted to validate its effectiveness and assess long-term functional and economic benefits. With appropriate support and integration, SCODA could serve as a cornerstone for national prehabilitation policies across LMICs.

## Figures and Tables

**Table 1:** Baseline Characteristics and Preoperative Measures. Comparison of demographic and clinical characteristics between the total cohort, pre-SCODA, and SCODA groups. Values are presented as mean (SD), median (IQR), or *n* (%).

Variable	Total (*n* = 169)	Pre-SCODA (*n* = 83)	SCODA (*n* = 86)	*p*-Value (Univariate Analysis)
Age, mean (SD)	55.4 (9.9)	54.4 (10.5)	56.3 (9.1)	0.170
Sex (Male), n (%)	73 (43.2)	38 (45.8)	35 (40.7%)	0.480
BMI *, mean (SD)	22.7 (3.6)	23.4 (3.8)	22.0 (3.3)	0.090
PCI *, median (IQR)	10 (8–14)	10 (8–13)	10 (9–14)	0.410
ASA * score ≥ 3, n (%)	128 (75.7)	56 (67)	72 (84)	0.010
ECOG * 2–3, n (%)	62 (36.7)	28 (34)	34 (40)	0.390
Primary tumor CRC * GC * OvC * PMP *	81 (47) 7 (4) 42 (24) 39 (23)	43 (51) 3 (3.6) 18 (21.6) 19 (22.9)	38 (44) 4 (4.6) 24 (28) 20 (23)	0.730
Neoadjuvant therapy, n (%)	51 (30.2%)	22 (23%)	29 (33%)	0.170
CC2 * resection	10	6 (7.2)	4 (4.5)	0.530
type of procedure CRS * only CRS + HIPEC *	134 35	72 (86.7) 11 (13.3)	62 (72) 24 (28)	0.023
Number of anastomoses > 2, n (%)	41 (24.3%)	21 (25.3%)	20 (23.3%)	0.780
Preoperative hemoglobin, mean (SD)	10.9 (1.5)	10.2 (1.4)	11.5 (1.2)	<0.001
Preoperative albumin, mean (SD)	3.5 (0.3)	3.4 (0.4)	3.5 (0.3)	0.140

* BMI: body mass index, ECOG: Eastern Cooperative Oncology Group Performance Status (determines the ability of a patient to tolerate therapies in serious illness, specifically for chemotherapy). ASA: American Society of Anesthesiologists Classification, CRC: Colorectal Cancer, GC: Gastric Cancer, OvC: Ovarian Cancer, PMP: Pseudomyxoma Peritonei, CC: Completeness of Cytoreduction score; CC2: indicates residual tumor nodules between 2.5 mm and 2.5 cm in diameter, representing incomplete cytoreduction. CRS: Cytoreductive Surgery. HIPEC: Hyperthermic intraperitoneal chemotherapy. PCI: Peritoneal Cancer Index.

**Table 2:** Perioperative Outcomes and SCODA-Specific Measures. Comparison of key postoperative outcomes and adherence rates between pre-SCODA and SCODA groups. SCODA-specific adherence data are available only for the SCODA group.

Variable	Pre-SCODA (n = 83)	SCODA (n = 86)	*p*-Value
Pulmonary complications, n (%)	11 (13%)	2 (2%)	0.004
Major complications (CD * ≥ 3b), n (%)	17 (21%)	9 (10.5%)	0.030
Post-op ICU * stay > 3 days, n (%)	32 (38.6%)	14 (16.3%)	0.010
Immediate post-op transfusion requirements, n (%)	17 (20%)	7 (8%)	0.010
90-day mortality, n (%)	10 (12.4%)	5 (5.8%)	0.040
Postoperative hemoglobin, mean (SD *)	9.2 (1.3)	10.6 (1.1)	<0.001
Postoperative albumin, mean (SD)	2.9 (0.3)	3.1 (0.4)	0.090
ECOG * 2–3 at discharge, n (%)	30 (36%)	19 (22%)	0.030
SCODA adherence to iron supplementation, n (%)	-	73 (85%)	-
SCODA adherence to physical activity, n (%)	-	69 (80%)	-
SCODA adherence to protein intake, n (%)	-	76 (88%)	-

* CD ≥ 3b: Clavien–Dindo ≥ 3b, Post-op ICU: post operative intensive care unit, SD: standard deviation, ECOG: Eastern Cooperative Oncology Group Performance Status (determines the ability of a patient to tolerate therapies in serious illness, specifically for chemotherapy).

**Table 3:** Multivariate analysis of different outcomes.

Outcome	Variable	Adjusted OR (95% CI)	*p*-Value
Pulmonary Complications	SCODA Group (yes)	0.26 (0.1–0.64)	0.004
	PCI * (per unit)	1.21 (1.08–1.36)	0.001
Major Complications (CD3b *)	SCODA Group (yes)	0.56 (0.28–1.13)	0.106
	PCI (per unit)	1.15 (1.05–1.26)	0.002
	Age (per year)	1.04 (1–1.08)	0.043
Transfusion Requirement	SCODA Group (yes)	0.16 (0.07–0.38)	0.005
	PCI (per unit)	1.13 (1.03–1.25)	0.014
	Preop Hb * (per g/dL)	1.1 (0.86–1.43)	0.415
ICU * Stay > 3 Days	SCODA Group (yes)	0.36 (0.16–0.81)	0.014
	PCI (per unit)	1.2 (1.08–1.33)	0.001
	ECOG 2–3 *	1.95 (1.31–2.9)	0.001
90-Day Mortality	SCODA Group (yes)	0.41 (0.20–0.84)	0.014
	PCI (per unit)	1.19 (1.09–1.31)	0.005

* PCI: Peritoneal Cancer Index, CD3b: Clavien–Dindo ≥ 3b, ICU: Intensive care unit, ECOG: Eastern Cooperative Oncology Group Performance Status. Preop Hb: pre-operative hemoglobin.

## Data Availability

The data presented in this study are available on request from the corresponding author. The data are not publicly available due to privacy restrictions.

## Supplementary Materials

The following supporting information can be downloaded at: https://www.mdpi.com/article/10.3390/cancers17223687/s1, Table S1: Tumor staging, histology, and surgical characteristics by group (Pre-SCODA vs. SCODA).
