# Supplementary material for: SCODA: A Low-Cost Prehabilitation Strategy to Improve Outcomes After Cytoreductive Surgery in a Low-Resource Setting
Source: Cancers (Basel). 2025 Nov 18;17(22):3687. doi: 10.3390/cancers17223687 (PMC12651516; doi:10.3390/cancers17223687)
Supplement: Supplementary file 1 [file cancers-17-03687-s001.zip › cancers-3825530-supplementary.pdf]

Supplementary Table. Tumor Staging, Histology, and Surgical Characteristics by Group (Pre-SCODA vs SCODA)

Variable	Pre-SCODA (n = 83)	SCODA (n = 86)
Primary Tumor Origin		
– Colorectal	43 (51)	38 (44)
– Gastric	3 (3.6)	4 (4.6)
– Ovarian	18(21.6)	24 (28)
– Pseudomyxoma Peritonei	19 (22.9)	20 (23)
Histologic Type		
– Adenocarcinoma	50 (60)	48 (55.8)
– High-grade serous carcinoma	14 (16.9%)	18 (20.9%)
– Mucinous neoplasm	19 (22.9)	20 (20.9%)
Peritoneal Cancer Index (PCI)		
– Median PCI [IQR]	10 [8–13]	10 [9–14]
Surgical Procedure Type		
– >4 regions peritonectomy	15 (18)	17 (20.5)
– Proctectomy	13 (15.6)	12 (13.9)
– Gastrectomy	4 (4.8)	5 (5.8)
– Spleno-pancreatectomy	5 (6)	4 (4.6)
– Bowel Anastomoses >2	21 (25.3)	20 (23.2)
– Urologic procedures	0 (0)	2 (2.3)
CC Score (Completeness of CRS)		
– CC-0	62 (74.7%)	71 (82.6%)
– CC-1	15 (18.1%)	11 (12.8%)
– CC-2	6 (7.2)	4 (4.5)
Staging (when applicable)		
– Stage IIIB–IV OvC (n = 38 total)	17 (100%)	21 (100%)
